# An evaluation of nutritional impact symptoms and their association with reduced dietary intake in patients with solid tumors at tertiary care hospitals: a multicenter, cross-sectional study from Palestine

**DOI:** 10.1186/s12885-024-12289-4

**Published:** 2024-04-25

**Authors:** Muna H. Shakhshir, Husam T. Salameh, Riad Amer, Sa’ed H. Zyoud

**Affiliations:** 1 Department of Nutrition, An-Najah National University Hospital, Nablus, 44839 Palestine; 2 Department of Public Health, College of Medicine and Health Sciences, An-Najah National University, Nablus, 44839 Palestine; 3 Department of Hematology and Oncology, An-Najah National University Hospital, Nablus, 44839 Palestine; 4 Department of Medicine, College of Medicine and Health Sciences, An-Najah National University, Nablus, 44839 Palestine; 5 Department of Clinical and Community Pharmacy, College of Medicine and Health Sciences, An-Najah National University, Nablus, 44839 Palestine; 6 Clinical Research Center, An-Najah National University Hospital, Nablus, 44839 Palestine

**Keywords:** Nutritional impact symptoms, Dietary intake, Solid cancer, Malnutrition, Palestine

## Abstract

**Background:**

Nutritional impact symptoms (NISs) are proposed to be a key indicator of decreased dietary intake in patients with solid cancer. Cancer patients frequently experience NIS from the disease itself and from disease treatment side effects that impact oral and gastrointestinal health. Thus, this study aimed to investigate the association between NIS and dietary intake among cancer patients in the Nablus district, one of the largest districts in Palestine. This study also sought to identify the types of treatment and other factors related to dietary intake for solid cancer patients.

**Methods:**

A cross-sectional study was conducted between October 15, 2021, and October 15, 2022. The convenience sampling technique was used to recruit participants from two primary hospital campuses for cancer treatment in the entire region of Nablus Governorate in northern Palestine. To assess the patients, structured questionnaires completed by interviewers during face-to-face interviews with patients were used. The NIS was assessed using a checklist developed based on a literature review and clinical experience. Univariate and multivariate analyses were used to evaluate the correlations between sociodemographic variables and clinical variables and between the NIS and dietary intake. Multiple binary logistic regression analyses were also performed to determine the most influential variables, sociodemographic, clinical, and NIS, on dietary intake.

**Results:**

Data were collected from 290 patients with solid malignancies. The mean age of the participants was 55.04 ± 12.76 years. Multiple binary logistic regressions revealed that dry mouth (odds ratio (OR) = 3.742; 95% confidence interval (CI) = 1.800–7.780; *p* < 0.001), constipation (OR = 2.707; 95% CI = 1.113–6.581; *p* = 0.028), taste alteration (OR = 3.620; 95% CI = 1.752–7.481; *p* = 0.001), and feeling fullness (OR = 8.879; 95% CI = 2.982–26.441; *p* < 0.001) were significantly related to decreased dietary intake. Biological and hormonal treatments had an inverse association with dietary intake (OR = 0.372; 95% CI = 0.177–0.782; *p* = 0.009 and OR = 0.383; 95% CI = 0.168–0.874; *p* = 0.023, respectively).

**Conclusions:**

This study revealed that many solid cancer patients have reduced food intake due to NIS, such as dry mouth and taste changes. These patients may be at risk of malnutrition. Healthcare professionals should consider these NISs to improve dietary plans and decide whether extra feeding support is needed. The results obtained indicate the need for further research focused on removing limitations in food consumption as an effect of treatment and appropriate nutritional strategies to prevent patient malnutrition.

**Supplementary Information:**

The online version contains supplementary material available at 10.1186/s12885-024-12289-4.

## Background

Dietary intake is a crucial concept since all diet-health hypotheses and dietary interventions are meant to be followed over time to achieve the proposed outcomes and formulate nutritional policies and guidelines for individuals, groups, and communities [1, 2]. Therefore, the term usual dietary intake refers to the long-term average daily intake of a food or nutrient [3]. Adequate food and energy intake is a critical factor in determining hospital length of stay, cost, and patient clinical and nutritional status [4, 5].

The purpose of dietary intake assessment is vital when we wish to investigate the connections between nutrition and health or when we wish to know the distribution of usual intake for a population or subgroups in epidemiological studies [6, 7]. Thus, assessments of dietary intake vary from individual nutritional screening in clinical settings to the adequacy of intake by population groups for use in research relating to diet and health. There are many assessment tool dimensions for tracking dietary intake despite random and systematic measurement errors [6–8].

Sufficient oral intake is contingent upon good oral and gastrointestinal health [9]. The most important barriers that patients may not eat in hospitals include three main categories: food taste and aroma, patient pain and symptoms, and tray delivery systems [10]. Insufficient dietary consumption among hospitalized patients is a prevalent issue that can result in malnutrition, which is correlated with an elevated likelihood of prolonged hospitalization [11], readmission, infections, and other complications [12–14].

Cancer patients, ranging from 20% to over 70%, are particularly susceptible to malnutrition [15–17]. However, 10–20% of cancer patients die from malnutrition rather than the disease itself [18]. Malnutrition in cancer patients refers to a substantial loss of weight and bodily assets due to inadequate nutritional intake caused by a number of factors and is linked to a depletion of body fat and lean mass stores. These factors include changes in metabolism, cancer therapy, and the tumor itself, especially those that affect the gastrointestinal tract [19–21]. Moreover, individuals living with cancer and receiving cancer treatment often experience multiple sudden and stagy nutrition-related side effects that impact nutritional status and quality of life [22]. Thus, low meal intake represents an independent risk factor for hospital mortality [23].

Nutritional impact symptoms (NISs) are defined as a broad spectrum of barriers to oral dietary intake [24, 25]. Fourteen gastrointestinal-oral symptoms have been documented as the most common impediments to eating; these include difficulty swallowing, mouth sores, dry mouth, feeling full, constipation, diarrhea, anorexia, nausea, vomiting, changes in taste, smell, fatigue, pain, and other reasons [26]. These symptoms are potentially prevalent at the early stages of cancer and can affect dietary intake regardless of current nutritional status or caloric intake [25–27]. Previous studies have shown that inadequate nutrition and unhealthy eating habits can significantly increase the risk of developing oral diseases, including tooth decay (dental caries) and even cancer, particularly among older adults [28–30]. In contrast, few studies have investigated the effect of NIS, such as poor oral-gastrointestinal conditions, on reduced dietary intake in cancer patients, and the association is still not entirely evident [28, 31, 32]. In 2022, the total number of newly reported cancer cases in the West Bank was 3,408, with a crude incidence rate of 118.4 per 100,000 people. However, the cancer incidence rate in Nablus Governate alone was 126.3 per 100,000, making it the fifth highest incidence rate in Palestine. Cancer is the second leading cause of death in Palestine in 2022, with a mortality rate of 42.6 per 100,000 people. Breast cancer is the most common cancer, followed by colorectal and lung cancer [33]. Therefore, this article evaluated the associations of the NIS and other factors, particularly among solid cancer patients, with reduced dietary intake, as no previous studies have been conducted on the NIS as an independent risk factor for reducing dietary intake in patients with solid tumors receiving anticancer treatments.

## Methods

### Study design

This was a cross-sectional study. The data were collected between October 15, 2021, and October 15, 2022.

### Settings

The study collected data from patients with solid tumors treated with chemotherapy at two tertiary hospitals, An-Najah National University Hospital (NNUH) and Al-Watani Governmental Hospital, in the Nablus-West Bank-Palestine. The Governorate of Nablus, with 348,000 residents, is regarded as a significant business and cultural hub for the Palestinian people and consists of forty-three villages, eight towns, and three refugee camps.

The two mentioned campuses represent important cancer treatment facilities since they are the primary referral hospitals for the entire region.

### Sampling method and sample size calculation

According to the 2020 Annual Report of the Palestinian Ministry of Health, 495 individuals in Nablus were undergoing treatment for cancer [33]. The majority of these patients received care at two primary tertiary hospitals specializing in cancer treatment, offering comprehensive services, including chemotherapy and biological and hormonal therapy. These significant data played a pivotal role in determining the appropriate sample size for our research.

A convenience nonrandom sampling technique was used to achieve our research goals. The sample size was determined utilizing the Raosoft sample size calculator, an automated software tool accessible at http://www.raosoft.com/samplesize.html. This calculation was based on a 5% margin of error with a 95% confidence interval, a 50% response distribution, and a population of 495 participants. Initially, a sample size of 217 was identified as the minimum required for effectiveness within the two-hospital context. However, to enhance the representativeness of our findings for the broader population, we augmented the sample size to 290.

### Inclusion and exclusion criteria

The study included men and women who were 18 years of age or older, who gave their permission to participate, who were diagnosed with any type of solid tumor, who were diagnosed at all stages of cancer development, and who were receiving anti-cancer therapy. Individuals lacking post-discharge follow-up data, individuals with intellectual disabilities, and those diagnosed with blood cancer were not included.

### Data collection form

A questionnaire was used to investigate the association between the NIS and dietary intake among the participants, in which the patients declared whether dietary intake decreased compared to the previous month or not. The data collection instruments included three sections. The first section collected sociodemographic data (age, sex, marital status, etc.), the second section collected clinical information (comorbidities, cancer details, treatment, diet), and the third section assessed the NIS. The NIS was assessed using a checklist developed based on a literature review and clinical experience (Additional file 1). This NIS checklist was developed based on a literature review and benefited from previous related studies, Patient-Generated Subjective Global Assessment (PG-SGA tool), multi-professional clinical expert opinions, and clinical experience where the NIS that might have a significant effect on reduced dietary intake was mentioned [26, 27, 34–36].

### Validity and reliability

A standardized questionnaire that had been translated into Arabic was utilized to evaluate the patients. The final questionnaire was given in Arabic. To ensure the accuracy and effectiveness of the questionnaire, a multi-step validation process was employed [37]. First, academic and clinical experts reviewed the instrument for face and content validity. This involved assessing the questionnaire’s organization, medical terminology, and comprehensiveness and clarity. Based on their feedback, the questionnaire was refined. Pilot testing with 15 patients further ensured clarity and readability. While the pilot data were not included in the final analysis, participant feedback was used to finalize the questionnaire. The questionnaire focused on 12 items related to the NIS and dietary limitations caused by these symptoms. Two simple response options (“yes” or “no”) were provided. Face and content validity were established through expert review and extensive literature review. The internal consistency of the 12 NIS questions was evaluated using Cronbach’s alpha, which demonstrated good reliability (alpha = 0.719). Therefore, the implemented validation process yielded a reliable and valid tool for measuring the NIS in patients with solid tumors.

### Data collection procedure

After receiving authorization from the Ministry of Health’s Education Department and the Clinical Research Center at NNUH, we obtained access to the medical records of patients in each center to identify individuals with solid cancer. Patients who met our inclusion criteria were then invited to participate in the study. We conducted face-to-face interviews with two hundred ninety-eight patients, prioritizing this method to ensure comfort for both participants and interviewers. Sociodemographic and clinical data were collected directly from the patients and their medical records. The questionnaire was administered in person, taking approximately ten minutes to complete. Before the interviews, the researchers provided background information on the project and clarified the questionnaire queries. Participants received a consent form outlining the study’s objectives and ensuring confidentiality, with the freedom to decide on participation. A final sample of 290 patients was obtained. Following direct interactions with patients and in-person interviews, data were retrieved from each hospital’s patient information system.

### Statistical analysis

The data were managed and analyzed using IBM-SPSS (version 25), a statistical package for social sciences (SPSS, Inc., Chicago, IL, USA). Descriptive statistics were used to ascertain response frequencies and to characterize the sample. Categorical variables are represented in terms of frequencies and percentages. Differences in proportions were assessed using either Fisher’s exact test or the chi-square test, with a significance threshold set at *p* < 0.05. This study investigated the factors correlated with decreased dietary intake in cancer patients undergoing treatment utilizing multivariate analysis to explore the interplay between sociodemographic variables (independent), clinical characteristics (independent), NIS (independent), and dietary intake (dependent). Four distinct models were constructed: Model 1 examined the association between sociodemographic variables (gender, marital status, education level) and reduced dietary intake; Model 2 focused on the association between clinical characteristics (cancer diagnosis, treatment modality) and reduced dietary intake; Model 3 analyzed the link between nutritional impact symptoms (dry mouth, taste changes, fatigue, etc.) and reduced dietary intake. A final model amalgamated significant factors from all the first three models to ascertain the most influential predictors of reduced dietary intake. Statistical analysis was conducted using multiple binary logistic regression across all models to evaluate the simultaneous effect of multiple independent variables on reduced dietary intake, and the odds ratio (OR) and confidence interval (CI) were calculated to quantify the strength and significance of the associations. A statistically significant association was denoted by a *p* value less than 0.05. An OR greater than 1 suggested an increased likelihood of reduced dietary intake with the corresponding variable, while an OR less than 1 indicated a decreased likelihood of reduced dietary intake.

## Results

### Patient demographic characteristics

Two hundred ninety patients participated in the present study, resulting in a response rate of 97.3%. The patients’ ages ranged from 18 to 88 years, with a mean age of 55.04 ± 12.76 years and a median age of 56 years, accompanied by an interquartile range of 46.0 to 64.0 years. The majority of the participants were female (67.6%), aged 50 years or older (67.6%), married (69.7%), and unemployed (81.4%). Approximately half of the respondents resided in rural areas (50.3%), had a secondary level of education (48.3%), and reported a monthly income between 400 and 1000 JD (57.6%) (Table 1).

**Table 1:** Associations between sociodemographic characteristics and reduced dietary intake (*n* = 290)

Variables	Total (%) *n* = 290	Reduced dietary intake Yes *n* = 215 (%)	Reduced dietary intake No *n* = 75 (%)	*P* value^1^	Univariate analysis	Multivariate analysis (model 1)
					Odds ratio with 95% CI	Odds ratio with 95% CI ^5^
**Age Category (years)**						
18–29 30–39 40–49 50–59 60–69 ≥70	12 (4.1) 21 (7.2) 61 (21) 81 (27.9) 80 (27.6) 35 (12.1)	9 (4.2) 16 (7.4) 42 (19.5) 61 (28.4) 60 (27.9) 27 (12.6)	3 (4) 5 (6.7) 19 (25.3) 20 (26.7) 20 (26.7) 8 (10.7	0.946^2^	Ref. (1) 1.067 (0.205–5.543) 0.737 (0.179–3.032) 1.017 (0.250–4.126) 1.000 (0.246–4.060) 1.125 (0.244–5.177)	
**Gender**						
Female Male	196 (67.6) 94 (32.4)	79 (36.7) 136 (63.3)	15 (20) 60 (80)	**0.008** ^**3**^	Ref. (1) 0.430 (0.229–0.808)	Ref. (1) 0.724 (0.309–1.699)
**Marital status**						
Married Single, divorced, widowed	202 (69.7) 88 (30.3)	140 (48.3) 75 (34.9)	62 (82.7) 13 (17.3)	**0.004** ^**3**^	Ref. (1) 2.555 (1.320–4.945)	**Ref. (1)** **2.072 (1.017–4.221)**
**Educational level**						
No formal education Primary school High school University degree and above	16 (5.5) 69 (23.8) 140 (48.3) 65 (22.4)	14 (6.5) 57 (26.5) 103 (47.9) 41 (19.1)	2 (2.7) 12 (16) 37 (49.3) 24 (32)	**0.041** ^2^	Ref. (1) 0.679 (0.136–3.385) 0.398 (0.086–1.834) 0.244 (0.051–1.167)	Ref. (1) 0.514 (0.080–3.293) 0.357 (0.061–2.093) 0.266 (0.043–1.628)
**Residency**						
Urban Rural Palestinian refugee Camp	128 (44.1) 146 (50.3) 16 (5.5)	92 (42.8) 111 (51.6) 12 (5.6)	36 (48) 35 (46.7) 4 (5.3)	0.733^2^	Ref. (1) 1.241 (0.722–2.132) 1.174 (0.355–3.880)	
**Professional status**						
Working Not working	54 (18.6) 236 (81.4)	37 (17.2) 178 (82.8)	17 (22.7) 58 (77.3)	0.296^3^	1.410 (0.739–2.691) Ref. (1)	
**Monthly average income** ^4^						
Less than 400 JD 400–1000 JD ≥ 1000 JD	81 (27.9) 167 (57.6) 42 (14.5)	64 (28.8) 117 (54.4) 34 (15.8)	17 (22.7) 50 (66.7) 8 (10.7)	0.176^3^	Ref. (1) 0.622 (0.331–1.166) 1.129 (0.442–2.883)	

JD: Jordanian Dinar (1 JD equals 1.41 US dollars)

^1^ Bold values denote statistical significance at the level of *p* < 0.05

^2^ Statistical significance of differences calculated using Fisher’s exact test

^3^Statistically significant differences were calculated using the chi-square test

^4^Jordanian Dinar (JD) equals 1.41 US dollars

^5^Bold values for odds ratios with 95% CIs denote statistical significance at the *p* < 0.05 level in the multivariate analysis model

### Clinical characteristics of the patients

Among the participants, the most common comorbidities were hypertension (28.6%) and diabetes (23.8%). Breast cancer was the most prevalent cancer type (39.3%), followed by colorectal (26.2%) and lung cancer (10.3%). Chemotherapy was the most common treatment (76.6%), followed by biological (23.8%) and hormonal (15.5%) therapies. Surgery was less frequent (8.3%), and only 3.1% of the patients received radiotherapy. Most patients (51%) had been diagnosed for 1–5 years, 41% were newly diagnosed (less than a year), and 7.9% had been diagnosed for more than 5 years. As detailed in Table 2. A total of 74.4% of patients reported reduced dietary intake, while 25.6% maintained their usual intake.

**Table 2:** Associations between clinical determinants and reduced dietary intake

Variables	Total (%) *n* = 290	Reduced dietary intake Yes *n* = 215 (%)	Reduced dietary intake No *n* = 75 (%)	*P* value^1^	Univariate analysis	Multivariate analysis (model 2)
					Odds ratio with 95% CI	Odds ratio with 95% CI ^4^
**Comorbid diseases**						
**Hypertension**						
Yes No	83 (28.6) 207 (71.4)	65 (30.2) 150 (69.8)	18 (24) 57 (76)	0.304^2^	1.372 (0.750–2.512) Ref. (1)	
**Diabetes mellitus**						
Yes No	69 (23.8) 221 (76.2)	50 (23.3) 165 (76.7)	19 (25.3) 56 (74.7)	0.716^2^	0.893 (0.486–1.642) Ref. (1)	
**Type of solid cancer**						
Breast cancer						
Yes No	114 (39.3) 176 (60.7)	73 (34) 142 (66)	41 (54.7) 34 (45.3)	**0.002** ^2^	2.346 (1.374–4.006) Ref. (1)	0.885 (0.419–1.868) Ref. (1)
Colorectal cancer						
Yes No	76 (26.2) 214 (73.8)	62 (28.8) 153 (71.2)	14 (18.7) 61 (81.3)	0.085^2^	0.566 (0.295–1.087) Ref. (1)	
Lung cancer						
Yes No	30 (10.3) 260 (89.7)	28 (13) 187 (87)	2 (2.7) 73 (97.3)	**0.006** ^3^	0.183 (0.043–0.788) Ref. (1)	2.306 (0.483–11.014) Ref. (1)
Prostate cancer						
Yes No	14 (4.8) 276 (95.2)	12 (5.6) 203 (94.4)	2 (2.7) 73 (97.3)	0.250^3^	0.463 (0.101–2.120) Ref. (1)	
Uterus cancer						
Yes No	11 (3.8) 279 (96.2)	8 (3.7) 207 (96.3)	2 (2.7) 73 (97.3)	0.498^3^	0.709 (0.147–3.415) Ref. (1)	
Gastric cancer						
Yes No	9 (3.1) 281 (96.9)	8 (3.7) 207 (96.3)	1 (1.3) 74 (98.7)	0.276^3^	0.350 (0.043–2.843) Ref. (1)	
Bone cancer						
Yes No	9 (3.1) 281 (96.9)	6 (2.8) 209 (72.2)	3 (4) 72 (96)	0.424^3^	1.451 (0.354–5.954) Ref. (1)	
Ovarian cancer						
Yes No	8 (2.8) 282 (97.2)	5 (2.3) 210 (97.7)	3 (4) 72 (96)	0.342^3^	1.750 (0.408–7.507) Ref. (1)	
**Others** ^5^						
Yes No	19 (6.6) 271 (93.4)	13 (6) 202 (94)	7 (9.3) 68 (90.7)	0.333^2^	0.625 (0.240–1.631) Ref. (1)	
**Duration of cancer**						
< 1 year 1–5 year > 5 years	119 (41) 148 (51) 23 (7.9)	87 (40.5) 115 (53.5) 13 (6)	32 (42.7) 33 (44) 10 (13.3)	0.092^2^	Ref. (1) 1.282 (0.732–2.245) 0.478 (0.191–1.198)	
**Type of cancer treatments**						
**Chemotherapy**						
Yes No	222 (76.6) 68 (23.4)	180 (83.7) 35 (16.3)	42 (56) 33 (44)	**< 0.001** ^2^	4.041 (2.257–7.233) Ref. (1)	1.422 (0.642–3.146) Ref. (1)
**Biological treatment**						
Yes No	69 (23.8) 221 (76.2)	42 (19.5) 173 (80.5)	27 (36) 48 (64)	**0.004** ^2^	0.432 (0.242–0.771) Ref. (1)	**0.454 (0.234–0.880)** **Ref. (1)**
**Hormonal treatment**						
Yes No	45 (15.5) 245 (84.5)	21(9.8) 194 (90.2)	24 (32) 50 (66.7)	**< 0.001** ^2^	0.216 (0.112–0.418) Ref. (1)	**0.340 (0.135–0.857)** **Ref. (1)**
**Radiation therapy**						
Yes No	9 (3.1) 281 (96.9)	7 (3.3) 208 (96.7)	2 (2.7) 73 (97.3)	0.576^3^	1.228 (0.250–6.047) Ref. (1)	
**Surgery**						
Yes No	24 (8.3) 266 (91.7)	13 (6) 202 (94)	11 (14.7) 64 (85.3)	**0.020** ^2^	0.374 (0.160–0.877) Ref. (1)	0.416 (0.156–1.108) Ref. (1)

^1^ Bold values denote statistical significance at the level of *p* < 0.05

^2^Statistically significant differences were calculated using the chi-square test

^3^Statistical significance of differences calculated using Fisher’s exact test

^4^Bold values for odds ratios with 95% CIs denote statistical significance at the *p* < 0.05 level in the multivariate analysis model

^5^“brain tumor, endocrine, bladder, liver, kidney, and skin cancer”

### NIS of the patients

The study identified dry mouth (59%) as the most prevalent symptom, followed by changes in taste (52.1%), fatigue (43.4%), feeling full (37.9%), and loss of appetite (30.3%). Additionally, constipation (29%) and various other symptoms, including mouth sores (22.8%), dizziness/headache (21%), nausea/vomiting (18.6%), swallowing problems (16.6%), chewing problems (13.8%), and diarrhea (10.7%), were reported. Table 3 shows the detailed data on nutritional impact symptoms.

**Table 3:** Associations between the NIS and reduced dietary intake

Variables	Total (%) *n* = 290	Reduced dietary intake Yes *n* = 215 (%)	Reduced dietary intake No *n* = 75 (%)	*P* value^1^	Univariate analysis	Multivariate analysis (model 3)
					Odds ratio with 95% CI	Odds ratio with 95% CI ^4^
**Nutritional Impact Symptoms (NIS)**						
**Swallowing problems**						
Yes No	48 (16.6) 242 (83.4)	47 (21.9) 168 (78.1)	1 (1.3) 74 (98.7)	**< 0.001** ^2^	20.702 (2.803-152.983) Ref. (1)	7.805 (0.926–65.799) Ref. (1)
**Chewing problems**						
Yes No	40 (13.8) 250 (86.2)	39 (18.1) 176 (81.9)	1 (1.3) 74(98.7)	**< 0.001** ^2^	16.398 (2.212-121.579) Ref. (1)	3.314 (0.364–30.216) Ref. (1)
**Dry mouth**						
Yes No	171 (59) 119 (41)	149 (69.3) 66 (30.7)	22 (29.3) 53 (70.7)	**< 0.001** ^3^	5.439 (3.059–9.669) Ref. (1)	**2.776 (1.322–5.829)** **Ref. (1)**
**Mouth sore**						
Yes No	66 (22.8) 224 (77.2)	58(27) 157(73)	8 (10.7) 67(89.3)	**0.004** ^3^	3.094 (1.401–6.835) Ref. (1)	1.774 (0.658–4.782) Ref. (1)
**Loss of appetite**						
Yes No	88 (30.3) 202 (69.7)	84 (39.1) 131(60.9)	4 (5.3) 71 (94.7)	**< 0.001** ^2^	11.382 (4.008–32.324) Ref. (1)	**5.263 (1.547–17.909)** **Ref. (1)**
**Fatigue**						
Yes No	126 (43.4) 164 (56.6)	106 (49.3) 109 (50.7)	20 (26.7) 55 (73.3)	**< 0.001** ^**3**^	2.674 (1.501–4.764) Ref. (1)	0.638 (0.269–1.514) Ref. (1)
**Diarrhea**						
Yes No	31 (10.7) 259 (89.3)	27 (12.6) 188 (87.4)	4 (5.3) 71(94.7)	0.081^2^	2.549 (0.861–7.544) Ref. (1)	
**Constipation**						
Yes No	84 (29) 206 (71)	75 (34.9) 140 (65.1)	9 (12) 66 (88)	**< 0.001** ^3^	3.929 (1.854–8.323) Ref. (1)	**3.615 (1.423–9.180)** **Ref. (1)**
**Nausea/Vomiting**						
Yes No	54 (18.6) 236 (81.4)	51 (23.7) 164 (76.3)	3 (4) 72 (96)	**< 0.001** ^2^	7.463 (2.255–24.703) Ref. (1)	**4.552 (1.073–19.317)** **Ref. (1)**
**Dizziness/Headache**						
Yes No	61 (21) 229 (79)	57 (26.5) 158 (73.5)	4 (5.3) 71 (49.7)	**< 0.001** ^2^	6.403 (2.237–18.331) Ref. (1)	2.474 (0.623–9.815) Ref. (1)
**Taste changes**						
Yes No	151 (52.1) 139 (47.9)	131 (60.9) 84 (39.1)	20 (26.7) 55 (73.3)	**< 0.001** ^**3**^	4.289 (2.400-7.664) Ref. (1)	**3.976 (1.859–8.503)** **Ref. (1)**
**Feeling fullness**						
Yes No	110 (37.9) 180 (62.1)	105 (48.8) 110 (51.2)	5 (6.7) 70 (93.3)	**< 0.001** ^**3**^	13.364 (5.190-34.412) Ref. (1)	**4.860 (1.577–14.972)** **Ref. (1)**

^1^Bold values denote statistical significance at the level of *p* < 0.05

^2^Statistical significance of differences calculated using Fisher’s exact test

^3^Statistically significant differences were calculated using the chi-square test

^4^ Bold values for odds ratios with 95% CIs denote statistical significance at the *p* < 0.05 level in the multivariate analysis model

### Associations between sociodemographic variables and reduced dietary intake

Significant associations were observed between dietary intake and participant demographics, including gender (*p* = 0.008), marital status (*p* = 0.004), and education level (*p* = 0.041). Notably, males, single individuals, and those with lower educational attainment reported lower dietary intake than did their counterparts. A detailed breakdown of these associations is presented in Table 1 (Model 1). Subsequent analyses employing multiple logistic regression corroborated these initial findings. Specifically, individuals who were single had a significantly greater likelihood (odds ratio [OR] = 2.072; 95% confidence interval [CI] = 1.017–4.221) of reporting reduced dietary intake than married participants.

### The association between clinical determinants and reduced dietary intake

The present study revealed that patients diagnosed with specific cancers and those receiving various treatment modalities exhibited significant alterations in dietary intake. Patients diagnosed with specific diagnoses and treatments exhibited significant differences in dietary intake. Patients who were diagnosed with breast cancer (*p* = 0.002) or lung cancer (*p* = 0.006) or who underwent a variety of treatment modalities, including chemotherapy (*p* = 0.001), biological therapy (*p* = 0.004), hormonal therapy (*p* = 0.001), or even surgery (*p* = 0.02), exhibited significant differences in their decreased dietary intake. Notably, the duration of cancer and other cancer types did not show any statistically significant associations with dietary intake (details in Table 2). Further logistic regression analysis corroborated these findings (Table 2: Model 2). Reduced dietary intake was the outcome variable, while diagnosis and treatment groups (breast cancer, lung cancer, chemotherapy, biological therapy, hormonal therapy, or surgery) were the independent variables. The analysis revealed that patients receiving biological therapy (OR = 0.454; 95% CI = 0.234–0.880) and hormonal therapy (OR = 0.340; 95% CI = 0.135–0.857) had a significantly lower risk of experiencing decreased dietary intake, suggesting that these therapies might have had a mitigating effect on decreased dietary intake.

### The association between NIS and reduced dietary intake

The present study revealed statistically significant associations between the NIS and reduced dietary intake in clinical settings. These symptoms included swallowing problems (*p* = 0.001), chewing problems (*p* = 0.001), dry mouth (*p* < 0.001), mouth soreness (*p* = 0.004), loss of appetite (*p* < 0.001), fatigue *(p* = 0.037), constipation (*p* = 0.001), nausea (*p* < 0.001), dizziness (*p* < 0.001), taste change (*p* = 0.001), and feelings of fullness (*p* < 0.001), as demonstrated in Table 3. Further examination using multiple binary logistic regression (Table 3: Model 3), with reduced dietary intake (yes versus no) as the dependent variable and the presence of the eleven NISs as independent variables, revealed significant associations. Specifically, dry mouth (OR = 2.776; 95% CI = 1.322–5.829), loss of appetite (OR = 5.263; 95% CI = 1.547–17.909), constipation (OR = 3.615; 95% CI = 1.423–9.180), nausea/vomiting (OR = 4.552; 95% CI = 1.073–19.317), taste changes (OR = 3.976; 95% CI = 1.859–8.503), and feelings of fullness (OR = 4.860; 95% CI = 1.577–14.972) were significantly linked to reduced dietary intake.

### Multiple regression analysis of characteristics associated with reduced dietary intake

Multiple binary logistic regression analyses were conducted to examine the association between reduced dietary intake (yes versus no) as the dependent variable and the significant factors identified in Models 1, 2, and 3 as independent variables. The analysis revealed that only experiencing dry mouth (OR = 3.742; 95% CI = 1.800–7.780; *p* < 0.001), experiencing constipation (OR = 2.707; 95% CI = 1.113–6.581; *p* = 0.028), experiencing taste changes (OR = 3.620; 95% CI = 1.752–7.481; *p* = 0.001), and feeling fullness (OR = 8.879; 95% CI = 2.982–26.441; *p* < 0.001) were significantly associated with reduced dietary intake. Notably, the analysis also revealed that patients who underwent biologic therapy (OR = 0.372, 95% CI = 0.177–0.782, *p* = 0.009) or hormonal therapy (OR = 0.383, 95% CI = 0.168–0.874, *p* = 0.009) were significantly less likely to experience reduced dietary intake. These findings are summarized in Table 4, which presents the results of the multiple binary logistic regression model.

**Table 4:** Patient characteristics associated with reduced dietary intake according to the multiple binary logistic regression model

Variables	B	S.E.	Wald	*P* value ^1^	Odds ratio with 95% CI
**Dry mouth**	1.320	0.373	12.484	**0.000**	3.742 (1.800–7.780)
**Loss of appetite**	1.216	0.631	3.707	0.054	3.373 (0.978–11.628)
**Constipation**	0.996	0.453	4.824	**0.028**	2.707 (1.113–6.581)
**Nausea and vomiting**	0.881	0.737	1.426	0.232	2.412 (0.569–10.233)
**Changing in taste**	1.287	0.370	12.072	**0.001**	3.620 (1.752–7.481)
**Feeling fullness**	2.184	0.557	15.385	**0.000**	8.879 (2.982–26.441)
**Biological therapy**	-0.990	0.380	6.799	**0.009**	0.372 (0.177–0.782)
**Hormonal therapy**	-0.961	0.421	5.203	**0.023**	0.383 (0.168–0.874)
**Marital status**	0.379	0.423	0.801	0.371	1.461 (0.637–3.350)
**Constant**	-0.603	0.336	3.228	0.072	0.547

^1^ Significant associations are indicated by bold values at a significance level of *p* < 0.05

B: This typically represents the coefficient estimates. These coefficients indicate the magnitude and direction of the association between the predictor variables and the outcome variable

S.E: Standard errors are measures of the variability of the coefficient estimates

Wald: This refers to the Wald statistic, which is used to test the significance of individual coefficients in the logistic regression model

Odds ratio with 95% CI: The odds ratio (OR) is a measure of the association between an exposure and an outcome. The 95% confidence interval (CI) around the odds ratio provides a range of values within which we can be 95% confident that the true odds ratio lies. An OR greater than 1 suggested an increased likelihood of reduced dietary intake with the corresponding variable, while an OR less than 1 indicated a decreased likelihood of reduced dietary intake

## Discussion

The present study revealed statistically significant associations between the NIS and reduced dietary intake in clinical settings. The findings indicate a high prevalence of reduced dietary intake, primarily attributed to symptoms such as dry mouth, constipation and taste changes, which are significantly associated with reduced dietary intake. According to logistic regression analysis, further research in 2020 revealed that these symptoms increase the risk of malnutrition [25]. Early satiety is the sensation of being full after eating little—possibly just a few bites—caused by cancer or cancer treatment. The results of this study showed that feeling full is also an independent risk factor for reduced dietary intake. Similar results were observed by Galindo et al. in their study and in a multicenter study encompassing 4783 cancer patients, which revealed a correlation between poor nutritional status and poor prognosis with the presence of early satiety [38, 39].

The most obvious finding to emerge from our analysis is that good and functional oral health is essential for maintaining sufficient oral dietary intake among cancer patients receiving anticancer therapy. The study revealed that patients with constipation, dry mouth sensations and taste changes had poorer dietary intake. These results are in line with other studies showing that saliva facilitates meal dilution and transportation as well as the delivery of taste compounds to taste receptors, all of which are critical functions of saliva in the chewing, swallowing, and digestion processes. As such, saliva plays a major role in the continuous preservation and protection of taste receptors. Hence, decreased salivary production has a direct impact on taste perception [40].

Dry mouth or xerostomia can develop as a result of hyposalivation when unstimulated whole saliva flow rates are less than 0.1–0.2 ml/min [41]. Dry mouth and taste changes are strongly related to each other and have been reported to occur more frequently together than dry mouth occurring alone in many previous studies [9, 42]. Consistent with the literature, this research matches those obtained in earlier studies and revealed that dry mouth is the most common symptom affecting 59% of solid cancer patients, followed by taste impairments, which affect 52.1% of the same group. A scoping analysis revealed that taste impairment (17.6–93%) and dry mouth/xerostomia (40.4–93%) are two of the most common symptoms experienced by cancer patients [43] and can affect dietary intake through reduced taste sensitivity [9]. On the other hand, reduced secretion of saliva causes food to adhere to the oral mucosa instead of producing a bolus [44], increasing the possibility of dental caries, which can lead to tooth loss, as shown in patients with head and neck cancer who have received radiation treatment. Thus, xerostomia, which can cause taste alterations and sensitive oral mucosa, might negatively affect dietary intake and nutritional status in diseased and/or hospitalized patients. The most clinically relevant findings in our study were that dry mouth, taste changes, feelings of fullness, and constipation are independent risk variables for reduced dietary intake in cancer patients. Therefore, people who have xerostomia exhibit changes in their dietary preferences, and their diets must be adjusted accordingly to avoid sticky items. A recent review further supported these findings. This study highlighted the scarcity of research on dietary recommendations for patients with xerostomia [45].

On the other hand, diarrhea is an NIS that may have a major impact on nutritional intake, as it is extremely common in cancer patients, with 20–70% of patients receiving radiotherapy and between 50 and 80% receiving chemotherapy [46]. However, the yields in this investigation were lower than those in other studies, as only 10.7% of the participants experienced diarrhea, and diarrhea did not seem to be significantly correlated with reduced dietary intake due to many possible factors, including the type of cancer, stage of cancer, kind of treatment received, and delayed symptoms that may manifest weeks or months later [47, 48]. Furthermore, the bulk of the sample group in our study consisted of patients who had a disease duration of less than 5 years and who actually may have experienced acute diarrhea caused by therapy with fast-acting treatment and who were appropriately diagnosed and managed. In addition, longer hospital stays are associated with invasive procedures such as surgeries, a higher incidence of infection, and late-stage cancer that can induce severe diarrhea [49–52]; however, this may not apply to the patients in our sample, which included outpatient chemotherapy centers in the same hospitals. This highlights the need for more research on the specific causes of diarrhea and its optimal management. Using a multiple binary logistic regression model, this study revealed that dry mouth, taste changes, constipation, and feeling full are independent risk factors for reduced dietary intake in cancer patients.

Biological therapy or immunotherapy is a recent type of cancer treatment in which the body’s immune system is used or in a laboratory to boost the immune system, help the body target and fight cancerous cells or lessen the side effects of treatment [53–55]. Hormone therapy is a nontoxic therapy that includes drugs that control the body’s hormone levels to affect tumor growth in patients with hormone-sensitive cancers, such as breast or prostate cancer. It is used to reduce the size of the tumor or the risk of reoccurrence [56]. In contrast, conventional cancer treatments, such as chemical treatment and radiotherapy, utilize chemicals to destroy existing cancer cells, resulting in adverse effects, such as symptoms of oral discomfort and additional GI complications, that can interfere with patients’ ability to eat and digest food [57]. Contrary to our expectations, this study revealed that patients who underwent biological or hormonal therapy were less likely to experience reduced dietary intake after the use of multiple binary logistic regression (OR = 0.372; 95% CI = 0.177–0.782; *p* = 0.009 and OR = 0.383; 95% CI = 0.168–0.874; *p* = 0.023, respectively). Therefore, we hypothesized that chemotherapy and invasive procedures are inversely correlated with dietary intake (OR = 4.001; 95% CI = 2.257–7.233; *p* < 0.001) using univariate analysis, which is consistent with previous literature [58, 59].

Future investigations into the reduced dietary intake of individuals suffering from NIS are needed. These investigations are crucial for ensuring both oral gastrointestinal health comfort and maintaining adequate dietary intake, especially for cancer patients of all ages and stages of the disease.

### Limitations

This study has limitations that may affect the generalizability and reliability of its findings. One limitation stems from the cross-sectional design, which captures data at a single point in time. This design makes it challenging to establish causal associations between variables. Without longitudinal data, it is difficult to determine the direction of these associations and observe changes over time. Another limitation concerns the convenience sampling method employed at the primary centers. This method, where participants are chosen based on accessibility, can introduce bias and limit the generalizability of the results. The sample may not accurately represent the entire population of interest, so caution is necessary when extrapolating these findings to other contexts. Another limitation regarding the study sample should be that the study sample was not homogeneous in terms of sex, age, type of cancer, or stage of cancer advancement. These factors may vary in terms of both the NIS and reduced dietary intake. Other weaknesses of the study, such as social support, housing and food insecurity, should be highlighted by the lack of additional data on the socioeconomic status of the participants. Socioeconomic status is a factor that significantly influences food choices and nutrient intake.

Finally, practical recruitment constraints often lead to smaller sample sizes, particularly when data are collected from a single district such as Nablus. Smaller samples have lower statistical power, making it harder to detect subtle or nuanced effects. Additionally, they are more susceptible to the influence of outliers, potentially leading to inaccurate or misleading conclusions. In conclusion, while this study provides valuable insights, it is crucial to consider these limitations when interpreting the results and drawing generalizable inferences.

## Conclusions

The findings indicate a high prevalence of reduced dietary intake, primarily attributed to oral and gastrointestinal symptoms related to dry mouth, constipation, taste changes, and feeling full, which were significantly associated with reduced dietary intake in patients receiving chemotherapy. Patients who underwent biological or hormonal therapy were less likely to experience reduced dietary intake. These discrepancies may have resulted from a failure to conduct a proper and thorough evaluation of oral and gastrointestinal health status in outpatient settings. The screening of NIS associated with reduced dietary intake could improve the ability to choose the most appropriate forms and methods of nutritional intake, sustaining oral feeding in cancer patients and decreasing the incidence of malnutrition-related morbidity and mortality. Further investigation is required to evaluate the association between reduced dietary intake and various forms of anticancer therapies.

### Recommendations

The results of this study indicate that the reduction of unnecessary restrictions on oral feeding and the improvement of dietary intake among cancer patients may be achieved by incorporating comprehensive dietary consultation, early dietary interventions, assessments of oral and gastrointestinal health and pharmacological therapy into palliative care. The findings of this study suggest that integrating thorough evaluations of oral and gastrointestinal health into palliative care could prove effective at reducing undue restrictions on oral feeding and enhancing dietary intake among cancer patients, considering the types of anticancer treatment used.

Early nutritional treatments can prevent, treat, and mitigate adverse consequences to reduce NIS and malnutrition-related mortality and morbidity. Thus, maximizing supportive therapy for the strict management of disorders affecting dietary intake is recommended. A sufficient number of dietitians and clinical pharmacists on duty will enable them to assess patients’ situations on a frequent and ongoing basis, promptly administer the most suitable nutritional intervention, and eventually assist patients in developing greater tolerance to anticancer medications. More research and long-term studies are required to better comprehend the long-term effects and develop specialized treatments for this patient population.

### Practical significance and future possible health implications

1. Additional data regarding socioeconomic characteristics that may affect food preferences and dietary intake are a potential area of focus since there is a lack of data on this topic in our research. Further clinical approaches may investigate the implementation of comprehensive socioeconomic programs that provide cancer patients with health care assistance, including prescription medications and dental care, as well as educational guidance, social support, and meal delivery.
2. Reduced food intake in cancer patients is associated with cancer types and treatments, including chemotherapy, surgery, and biological and hormonal therapy. In addition, a significant association was found between reduced dietary intake and breast and lung cancer. Subsequent clinical investigations may concentrate on creating interdisciplinary nutritional support groups that offer individualized and customized dietary therapies and approaches to mitigate symptoms resulting from problems. In addition, further investigation is required to evaluate the association between reduced dietary intake and various forms of anticancer therapies.
3. This study employed the most common nutritional impact symptom surveys, and the results indicated a significant association with reduced dietary intake. This information may assist healthcare providers in identifying patients who may be at risk of having low dietary intake. In light of the potential benefits, future clinical practice may investigate the incorporation of multidimensional assessment tools into routine care. By adopting comprehensive assessment protocols, healthcare professionals can gain a deeper understanding of patients’ overall needs, identify high-risk individuals, personalize interventions, and track treatment outcomes effectively.
4. Healthcare professionals responsible for creating health policies should be aware that solid cancer patients have reduced dietary intake due to the NIS. Understanding these symptoms is crucial for screening patients for NIS and oral-gastrointestinal health and tailoring interventions aimed at improving nutritional outcomes in cancer patients undergoing treatment. This approach will help them make better decisions about the best therapy and method strategies for consuming nutrients and assessing whether oral feeding alone can meet patients’ nutritional needs.
5. These findings show that additional studies are needed to reduce cancer treatment-related dietary intake and develop suitable nutritional plans to avoid patient malnutrition. In light of these factors, it is recommended that interventional studies be carried out to clarify the effects of nutritional complications, modified nutritional approaches with thorough evaluations of socioeconomics and clinical characteristics, and workable clinical, nutritional and pharmacological interventions targeted at maintaining oral feeding in cancer patients. These studies will also support advancements in the nutritional field and evidence-based clinical practice.

### Electronic supplementary material

Below is the link to the electronic supplementary material.

Supplementary Material 1

## Data Availability

To protect participant privacy, the datasets used in this study are available from the corresponding author upon reasonable request.

## Abbreviations

NIS: Nutritional impact syndrome
OR: Odds ratio
CI: Confidence interval
NNUH: An-Najah National University Hospital
PG-SGA: Patient-Generated Subjective Global Assessment
SPSS: Statistical Package for Social Sciences
JD: Jordanians Dinar
IRB: Institutional Review Board
BMI: Body mass index
