# Supplementary material for: An evaluation of nutritional impact symptoms and their association with reduced dietary intake in patients with solid tumors at tertiary care hospitals: a multicenter, cross-sectional study from Palestine
Source: BMC Cancer. 2024 Apr 25;24:524. doi: 10.1186/s12885-024-12289-4 (PMC11046763; doi:10.1186/s12885-024-12289-4)
Supplement: Supplementary file 1 — Supplementary Material 1 [file 12885_2024_12289_MOESM1_ESM.docx]

**Additional File 1: Data collection form.** This is the final version of the English version that was used to investigate the relationship between nutritional impact syndrome (NIS) and dietary intake among cancer patients in one of the largest cities in Palestine. This study also sought to identify the types of treatment and other factors related to dietary intake for solid cancer patients.

**English version**

*You are invited to participate in a research study about nutritional impact syndrome and dietary intake. This questionnaire should take about 10 minutes to complete. An interviewer will fill out the questionnaire based on your answer. Participation is voluntary, and responses will be kept anonymous. The results will be used solely for research purposes.*

**Data Collection Form**

1. **Patient socio-demographic data**
2. **Age in years: ………………………**
3. **Gender:**

1. Male 2. Female

1. **Marital Status:**

1. Married 2. Single/divorced/separated/widowed

1. **Educational status:**

1. No formal education 2. Primary school 3. High school

4. University degree and above

1. **Residence:**

1. Urban 2. Rural 3. Palestinian refugee camp

1. **Professional status:**

1. Working 2. Not working

1. **Monthly average income:**

1. Less than 400 JD 2. 400-1000 JD 3. ≥ 1000 JD

1. **Clinical information data**
2. **Major comorbidities:**

1. Hypertension 2. Diabetes 3. Heart disease

4. Others: specify……………...

1. **Type of cancer:**

1. Breast cancer 2. Colorectal cancer 3. Lung cancer 4. Prostate

5. Uterus 6. Gastric 7. Bone 8. Ovarian 9. Others: specify……………….

1. **Duration of cancer: ………………………………………………**
2. **Medications used: ………………………………………………**
3. **Type of treatment:**

1. Chemotherapy 2. Radiotherapy 3. Surgery 4. Biological therapy

5. Hormonal therapy

1. **Did your dietary intake decrease compared to the previous month?**
2. Yes 2. No
3. **Nutritional impact symptoms**

| **Do you have the following nutritional difficulties:** | **Yes** | **No** |
| --- | --- | --- |
| 1. Swallowing problems |  |  |
| 1. Chewing problems |  |  |
| 1. Mouth sore |  |  |
| 1. Dry mouth |  |  |
| 1. Loss of appetite |  |  |
| 1. Fatigue |  |  |
| 1. Diarrhea |  |  |
| 1. Constipation |  |  |
| 1. Nausea/ vomiting |  |  |
| 1. Dizziness/headache |  |  |
| 1. Changes in taste |  |  |
| 1. Feeling fullness |  |  |
